# Convergence Behavior of DNNs with Mutual-Information-Based Regularization

**DOI:** 10.3390/e22070727

**Published:** 2020-06-30

**Authors:** Hlynur Jónsson, Giovanni Cherubini, Evangelos Eleftheriou

**Affiliations:** IBM Research Zurich, 8803 Rüschlikon, Switzerland; hlynur4@gmail.com (H.J.); ele@zurich.ibm.com (E.E.)

**Keywords:** deep neural networks, information bottleneck, regularization methods

## Abstract

Information theory concepts are leveraged with the goal of better understanding and improving Deep Neural Networks (DNNs). The information plane of neural networks describes the behavior during training of the mutual information at various depths between input/output and hidden-layer variables. Previous analysis revealed that most of the training epochs are spent on compressing the input, in some networks where finiteness of the mutual information can be established. However, the estimation of mutual information is nontrivial for high-dimensional continuous random variables. Therefore, the computation of the mutual information for DNNs and its visualization on the information plane mostly focused on low-complexity fully connected networks. In fact, even the existence of the compression phase in complex DNNs has been questioned and viewed as an open problem. In this paper, we present the convergence of mutual information on the information plane for a high-dimensional VGG-16 Convolutional Neural Network (CNN) by resorting to Mutual Information Neural Estimation (MINE), thus confirming and extending the results obtained with low-dimensional fully connected networks. Furthermore, we demonstrate the benefits of regularizing a network, especially for a large number of training epochs, by adopting mutual information estimates as additional terms in the loss function characteristic of the network. Experimental results show that the regularization stabilizes the test accuracy and significantly reduces its variance.

## 1. Introduction

Deep Neural Networks (DNNs) have revolutionized several application domains of machine learning, including computer vision, natural language processing and recommender systems. Despite their success, the internal learning process of these networks is still an active field of research. One of the goals of this paper is to leverage information theoretical concepts to analyze and further improve DNNs. The analysis of DNNs through the information plane, i.e., the plane of mutual information values that each layer preserves at various learning stages on the input and the output random variables, was proposed in [1,2]. Previous approaches for the visualization of the information plane applied non-parametric estimation methods that do not work well with high dimensional data [2,3,4], as in this case the estimation of mutual information is nontrivial. The information plane for small fully connected networks was visualized in [2]. The results in [2] suggested that most of the training epochs of a DNN, the ”compression phase”, are spent on compressing the input variables after an initial “fitting phase”. The existence of the compression phase was later questioned in [4,5,6,7], in case the finiteness of the mutual information between input/output and hidden-layer variables cannot be established. In this paper, we focus on Convolutional Neural Networks (CNNs) with high complexity. After briefly discussing non-parametric mutual information estimation methods, we present the convergence of mutual information on the information plane for a high-dimensional VGG-16 CNN [8] by resorting to Mutual Information Neural Estimation (MINE) [9]. The compression phase is evident from the obtained results, which confirm and extend the results previously found with low-dimensional fully connected networks, where methods with lower computational complexity were adopted to study the convergence in the information plane.

Furthermore, we consider DNNs with mutual-information-based regularization. The use of the mutual information between the input and a hidden layer of a DNN as a regularizer was suggested in [9,10,11]. The idea is based on the Information Bottleneck (IB) approach [12], which provides a maximally compressed version of the input random variable, while still retaining as much information as possible on a relevant random variable. Here we compare the accuracy achieved by a VGG-16 CNN, using well-known regularization techniques, such as dropout, batch normalization and data augmentation, with that of the same VGG-16 network that is enhanced by applying mutual-information-based regularization, by resorting either to MINE or the variational information bottleneck (VIB) technique [10], and demonstrate the advantages of mutual-information-based regularization, especially for a large number of training epochs.

The remainder of the paper is structured as follows. Basic definitions of mutual information and the formulation of the IB approach are recalled in Section 2. Non-parametric methods for the estimation of mutual information in DNNs are addressed in Section 3. The visualization of DNN convergence on the information plane using MINE is described in Section 4, whereas the advantages of long-term DNN regularization using mutual-information-based techniques are presented in Section 5. Finally, conclusions are given in Section 6.

## 2. The Information Bottleneck in DNNs

The mutual information is a measure of the mutual dependence of two random variables. In essence, it measures the relationship between them and may be regarded as the reduction in uncertainty in a random variable given the knowledge available about another one. The mutual information between two discrete random variables *X* and *Y* is defined as

(1) $$I\left(X;Y\right)=\sum_{x\in X}\sum_{y\in Y}p\left(x,\;y\right)\log\frac{p(x,\;y)}{p(x)p(y)}$$

where p(x, y) is the joint probability distribution and p(x) and p(y) are the marginal probability distributions. The mutual information may also be expressed as the Kullback–Leibler (KL) divergence between the joint distribution and the product of the marginal distributions of the random variables *X* and *Y*

(2) $$I\left(X;Y\right)=D_{KL}\left(p(x,\;y)∥p(x)p(y)\right)$$

Computing the mutual information of two random variables is in general challenging. Exact computation can be done in the discrete setting, where the sum in Equation (1) can be calculated exhaustively, or in the continuous setting, provided the probability distributions are known in advance. Several methods to estimate the mutual information have been introduced. The most common ones are non-parametric, including discretizing the values using binning and resorting to non-parametric kernel-density estimators, as will be discussed in more detail in Section 3.

The IB method was introduced in [12] to find a maximally compressed representation of an input random variable, *X*, which is obtained as a function of a relevant random variable, Ψ, such that it preserves as much information as possible on Ψ. Let us denote $\hat{X}$ as the compressed version of the input random variable *X* parameterized by θ. What the IB method effectively does is to solve the following optimization problem

(3) $$\begin{array}{c} \min_{\theta }I_{\theta }\left(X;\hat{X}\right)\\ \mathrm{subject}\;\mathrm{to}:\;I_{\theta }\left(\hat{X};\Psi \right)=I_{c}\leq I\left(X;\Psi \right) \end{array}$$

where $I_{c}$ is the information constraint. In a non-deterministic classification scenario, an equivalent formulation of the IB problem is obtained by introducing the Lagrangian multiplier $\beta^{\prime }\geq 0$, and by minimizing the Lagrangian

(4) $$L\left(\theta \right)=I_{\theta }\left(X;\hat{X}\right)-\beta^{\prime }I_{\theta }\left(\hat{X};\Psi \right)$$

In a deterministic classification scenario, however, where neural networks do not exhibit any randomization of the hidden layer output variables, the two IB formulations given above are in general not equivalent, as demonstrated in [13].

The goal of any supervised learning method is to efficiently capture the relevant information about an input random variable, typically a label for classification tasks, in the output random variable [1]. Let us consider a DNN for image recognition, with input *X*, output $\hat{Y}$, and *i*-th hidden layer denoted by $h_{i}$. The classification task is related to the interpretation of an image that is generated from a relevant random variable *Y*.

In case the hidden layer, $h_{i}$, only processes the output of the previous layer, $h_{i-1}$, the layers form a Markov chain of successive internal representations of the input. By applying the Data Processing Inequality (DPI) [14] to a DNN that consists of *L* layers, we have

(5) $$I\left(X;\;h_{1}\right)\geq I\left(X;\;h_{2}\right)\geq \ldots \geq I\left(X;\;h_{L}\right)\geq I\left(X;\hat{Y}\right)$$

As mentioned in the Introduction, DNNs may be analyzed through mutual information values on the information plane. However, estimating mutual information across layers in DNNs is a nontrivial task, as the outputs of the hidden layer neurons are continuous-valued high-dimensional random vectors.

A further difficulty arises if the input X is a continuous random variable and the neural network is deterministic. In this case, it has been shown, e.g., in [4,5,6], that $I(X;\;h_{i})=\infty$ for commonly used activation functions. For the image classification problem considered here, one might argue that the input random variable is a discrete random variable, where the pixels have a discrete distribution, and $h_{i}$ is also a discrete random variable given by a deterministic transformation of *X* via finite-precision arithmetic. The training and test sets, however, have a cardinality that is typically much lower than the alphabet size of *X*, thus rendering the estimation of the mutual information very difficult. To cope with the challenge of estimating the divergence in Equation (2), we will resort to MINE, as discussed in Section 4.

## 3. Non-Parametric Estimation of Mutual Information

**Figure 1:**
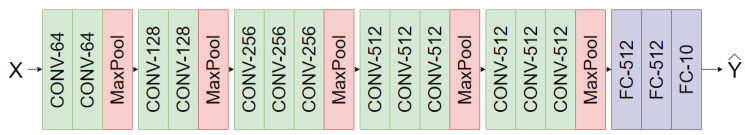
The Visual Geometry Group (VGG)-16 network [8] architecture from input to predicted output. CONV-64 is shorthand for a 2D convolutional layer with 64 filters. FC-512 is shorthand for a fully connected layer with 512 neurons.

As stated in Section 2, the most common methods for the estimation of mutual information are non-parametric. As our focus is on CNNs, we consider a VGG-16 network [8] to evaluate the effectiveness of non-parametric estimation methods. The block diagram of a VGG-16 network is illustrated in Figure 1. The loss function adopted to train the network is the cross-entropy loss obtained from the softmax output probabilities, that is

(6) $$L_{CE}=\sum_{m=1}^{M}y_{m}\log p_{m}$$

where $y_{m}$ is the binary value corresponding to the *m*-th class of a one-hot encoded vector defined by the class labels, $p_{m}$ is the softmax probability output for class *m*, and *M* is the number of classes in the classification task. In all experiments the dataset considered is CIFAR-10 [15], i.e., M=10, with a batch size of 128. CIFAR-10 is a dataset consisting of 60,000 images in 10 classes. Fifty thousand of those images are used for training and 10,000 for testing. Each image of the dataset is of size 32×32 with three color channels.

### 3.1. Activation Binning

The mutual-information estimation method adopted in [2] resorts to binning the activations of each layer into equal-sized bins. Binning ensures that the values are discretized, which allows to calculate the joint distribution and marginal distributions by counting the activations in each bin.

Despite the promising results reported in [2], this method has a number of limitations as a general method for mutual-information estimation. Firstly, the experiments in [2] were conducted using a fully connected network with only a few neurons, which has far fewer synaptic weights and neurons than typical CNN architectures. Another shortcoming is the usage of the *tanh* function as a non-linear activation function, which bounds the activations from −1 to 1. The ReLU activation function [16] is more commonly used and allows unbounded positive activations. This limitation is pointed out in [4], where the authors showed counterexamples of the compression phase using ReLU non-linear activations. Furthermore, in [2] the input is limited to a vector of 12 binary elements and the output is also binary. In this case binning is convenient because of the low number of dimensions of the input and output random variables.

We found that the method of binning the activations does not scale well with higher dimensionality. For example, we experimented with the activations of VGG-16 trained on CIFAR-10. For the classification of a CIFAR-10 image, the input random variable has a total of 3072 dimensions as opposed to 12 in [2]. Varying the number of bins where the activations are allocated was also found not to have a significant impact on the results. The mutual-information estimates for layers with high input dimensionality turned out not to satisfy the DPI. In addition, the estimates of both $I(X;\;h_{i})$ and $I(h_{i};Y)$ for the last few CNN layers converged to values close to zero. If $I(h_{i};Y)$ approximates zero the accuracy of the model should be roughly the same as for random guessing. This contrasts with the measured accuracy, which is higher than 90%, compared to 10% for random guessing.

### 3.2. Non-Parametric Kernel Density Estimation

We also conducted experiments on the kernel-density estimation method described in [4,17]. The assumption made is that the hidden layer activities are distributed as a mixture of Gaussian random variables with covariance matrix $\sigma^{2}I$. In [4], it is further assumed for analysis purposes that Gaussian noise is added to each hidden layer $T_{i}$, which is expressed as $T_{i}=h_{i}+\epsilon$, where $\epsilon ∼N(0;\;\sigma^{2}I)$. A mutual information upper bound with respect to the input is proposed as

(7) $$\begin{array}{c} I\left(T_{i};\;X\right)\leq -\frac{1}{P}\sum_{j}\log\frac{1}{P}\sum_{k}\exp\left(-\frac{1}{2}\frac{\left|\right|h_{ij}-h_{ik}{\left|\right|}_{2}^{2}}{\sigma^{2}}\right) \end{array}$$

where *P* denotes the number of samples and $h_{ij}$ the hidden layer activities of layer *i* for sample *j*. Furthermore, the mutual information with respect to the output random variable is upper bounded as follows

(8) $$\begin{array}{c} I\left(T_{i};\;Y\right)\leq -\frac{1}{P}\sum_{j}\log\frac{1}{P}\sum_{k}\exp\left(-\frac{1}{2}\frac{\left|\right|h_{ij}-h_{ik}{\left|\right|}_{2}^{2}}{\sigma^{2}}\right)\\ -\sum_{m}p_{m}\left[-\frac{1}{P_{m}}\sum_{j,Y_{j}=m}\log\frac{1}{P_{m}}\sum_{k,Y_{k}=m}\exp\left(-\frac{1}{2}\frac{\left|\right|h_{ij}-h_{ik}{\left|\right|}_{2}^{2}}{\sigma^{2}}\right)\right] \end{array}$$

where $P_{m}$ is the number of samples with class label *m*, and $p_{m}=P_{m}/P$ is the empirical probability of class *m*.

The same experiment as in [2] was conducted in [4] by using the non-parametric kernel density estimation.

We also tested the estimation method by a VGG-16 network, and adopted a variance of $\sigma^{2}=0.1$, as in [4]. However, as experienced with the binning method in Section 3.1, we did not find satisfactory results with a convolutional network of high complexity.

### 3.3. Rényi’s α-Entropy

A multivariate matrix-based Rényi’s α-entropy method was proposed in [3] for application to a LeNet-5 network. This approach is suitable for CNN networks, as each CNN layer has several channels, which all contain some information on the input and output random variables. However, two distinct channels of a single layer can contain the same information on the input and output random variables. Therefore, summing up the mutual information estimates between each channel and the input or output random variable only gives an upper bound on the mutual information that has little relevance to the true mutual information value. The experiments in [3] for LeNet-5 result in mutual information estimates for the various layers that satisfy the DPI. However, our experiments for VGG-16, which has up to 512 channels, did not yield estimates that comply with the DPI.

A method for the estimation of mutual information in complex DNNs was proposed in [18], which relies on matrix-based Rényi’s entropy and tensor kernels to estimate the mutual information in a VGG-16 network. The method in [18] augments the multivariate extension of the matrix-based Rényi’s α-order entropy presented in [19] by introducing tensor kernels. In that manner, the tensor-based nature of convolutional layers in DNNs is recognized and the numerical difficulties arising by a straightforward application of the multivariate extension of the matrix-based Rényi’s entropy are avoided. However, the convergence in the information plane is affected by the overfitting that takes place when the training is conducted for a large number of epochs. Therefore, the compression phase needs to be limited by an early stopping technique to prevent overfitting.

## 4. DNN Convergence Analysis Using MINE

Our goal is to visualize the information plane for networks with high-dimensional variables, as previous work focuses on networks with much lower complexity [2,4,10]. The methods discussed in Section 3 for estimating the mutual information do not perform well with high-dimensional random variables. Furthermore, the existence of a compression phase during training has been disputed in [4] for networks where the finiteness of the mutual information between input/output and hidden-layer variables cannot be established. Therefore, to clarify these issues, we consider a VGG-16 network with ReLU activation function. For the estimation of the mutual information for all layers in the network, we resort to the MINE method [9].

MINE is a method first proposed in [9] for the estimation of mutual information between high-dimensional continuous random variables. The method takes advantage of the Donsker–Varadhan dual representation of the KL-divergence [20] and utilizes the lower bound

(9) $$D_{KL}\left(P∥Q\right)\geq \underset{T\in F}{\sup}E_{P}\left[T\right]-\log\left(E_{Q}\left[e^{T}\right]\right)$$

where F is any class of functions $T:R^{d}\to R$ such that the two expectations are finite, and P and Q are probability distributions. The main idea of MINE is to choose *T* as a function parameterized by a deep neural network with parameters θ∈Θ. By defining P as the joint probability distribution and Q as the product of the marginal distributions of the random vectors *X* and *Y*, by combining Equations (9) and (2) we get the MINE lower bound

(10) $$\hat{I}\left(X;\;Y\right)=\underset{\theta \in \Theta }{\sup}E_{p(x,\;y)}\left[T_{\theta }\right]-\log\left(E_{p(x)p(y)}\left[e^{T_{\theta }}\right]\right)$$

The lower bound given by Equation (10) is finite, even if the input *X* is a continuous random vector and the neural network under investigation is deterministic, for which the mutual information between the input and a hidden layer $I(X;\;h_{i})$ is infinite, as discussed in Section 2. If the mutual information is not finite, the MINE may nevertheless be regarded as a well-defined estimate of the statistical divergence between the two probability distributions $p(X,\;h_{i})$ and $p\left(X\right)p\left(h_{i}\right)$ that assume nonzero (possibly infinite) values over different support. This is analogous to the evaluation of the optimal cost in applications of the optimal transport theory, which is obtained by resorting to a dual representation of the original problem, see, e.g., [21].

The expectation over the product of the marginal distributions is estimated by shuffling the samples from which the empirical joint distribution is obtained. For the estimation of $I(X;\;h_{i})$ the samples from $h_{i}$ are shuffled, whereas for the estimation of $I(h_{i};Y)$ the samples from *Y* are shuffled. The objective function from Equation (10) is adopted and is optimized by gradient ascent. For the visualization of the information plane for the *i*-th layer in the network, two estimations are needed, namely those of $I(X;\;h_{i})$ and $I(h_{i};Y)$. Each of these estimations is parameterized by a separate deep neural network. As stated in [9], more training samples are needed as the complexity of the MINE network increases. Therefore, very deep networks with high complexity are infeasible as MINE networks. Here we adopt a network and an overall training approach capable of accurately estimating mutual information while resorting to networks with moderate complexity.

**Figure 2:**
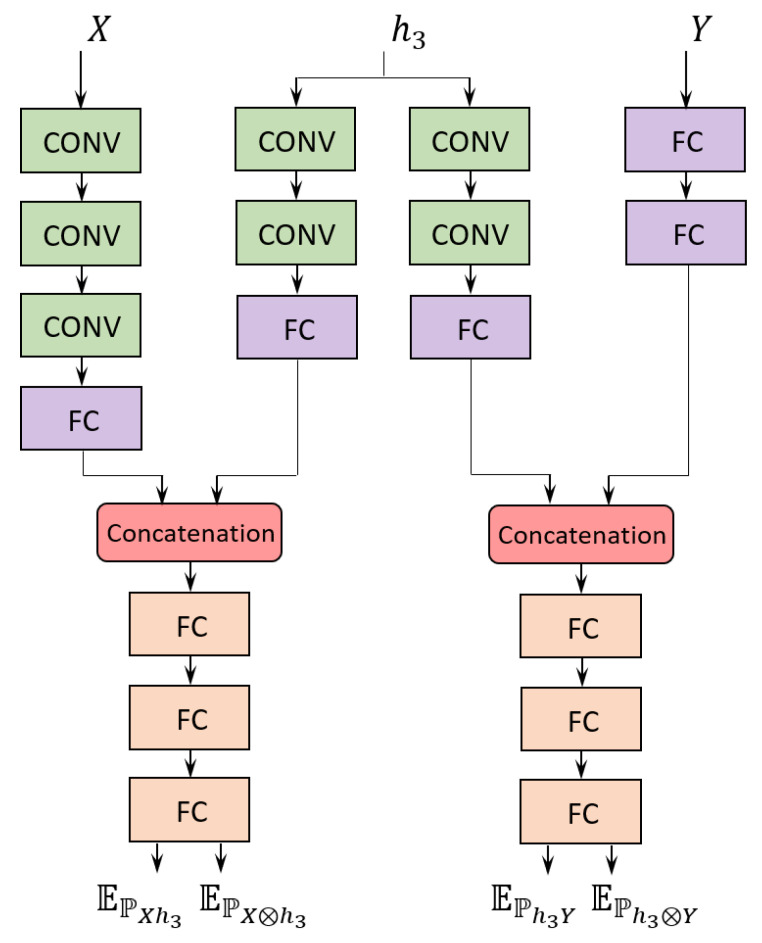
Example of the Mutual Information Neural Estimation (MINE) networks considered for both $I(X;\;h_{3})$ and $I(h_{3};\;Y)$ in layer 3 of VGG-16. The same input and output encoder are employed for all layers. The four expectations are applied as indicated in Equation (10) to estimate both $I(X;\;h_{3})$ and $I(h_{3};\;Y)$. FC indicates a fully connected layer, whereas CONV indicates a convolutional layer.

For the experiments in this section, we train a VGG-16 network on CIFAR-10 images. Minor data augmentation is applied in the form of random cropping and randomly flipping the images horizontally. The size of each CIFAR-10 image is 32×32 pixels. For random cropping, we pad the original image to 40 × 40 pixels and randomly take a crop of size 32 × 32 pixels. In our experiments, each convolutional layer has a 3×3 pixel receptive field. In addition, batch-normalization is used for all convolutional layers. Furthermore, dropout regularization is applied after all convolutional layers that do not precede a pooling layer (dropout rate of 0.3) and after the first fully connected layer (dropout rate of 0.5). The ReLU activation function is adopted for all layers with the exception of the last one, which is a linear dense layer. The hyperparameters are chosen using a validation set obtained by extracting 10,000 samples from the training set. The MINE loss function used to train each MINE network is defined as

(11) $$L_{MINE}=\frac{1}{n}\sum_{i=1}^{n}T_{\theta }\left(i\right)-\log\left(\frac{1}{n}\sum_{j=1}^{n}\exp^{T_{\theta }\left(j\right)}\right)$$

where *n* is the batch size and $T_{\theta }\left(i\right)$ and $T_{\theta }\left(j\right)$ are the individual network outputs referring to the expectation over the joint distribution and over the product of the marginal distributions (see Equation (10)), respectively. An illustration of the process, including input and output encoders and referring to the hidden layer $h_{3}$ as an example, is shown in Figure 2.

### 4.1. Visualization of the Information Plane

As discussed above with reference to Equation (10), each estimate of mutual information by MINE requires a separate neural network to learn both the expectations over the joint distribution and over the product of the marginal distributions. To characterize the convergence behavior of the VGG-16 on the information planes, we need to estimate both $I(X;\;h_{i})$ and $I(h_{i};Y)$ for each layer, i.e., a total of 32 networks are needed. Additionally, we adopt two encoders, which are employed across all layers, to encode the input and output random variables. Each MINE network encodes the respective hidden layer. As illustrated in Figure 2, all hidden layer encoders and input/output encoders output a 64-dimensional vector. The hidden layer encoder output is concatenated with the corresponding encoder output for the input/output random variable, resulting in a 128-dimensional vector. A fully connected network takes the concatenated vector as input and outputs a single value, from which the expected value over either distribution is obtained, depending on whether the input is shuffled or not. The expected values obtained from the network yield the mutual information estimate by Equation (10). Further details on the architectures of the experimental MINE networks are given in [22].

An information plane shows the mutual information estimates for all epochs within a certain layer. To get unbiased estimates for each epoch the training procedure is conducted as follows. Initially, the VGG-16 network is trained up to a certain epoch. Then all MINE networks are trained for a total of 1000 epochs. During MINE network training, the MINE networks use the outputs of the hidden layer neurons of the trained VGG-16 as input, without updating the weights of the VGG-16 through back-propagation. In this phase, gradient-ascent updating by back-propagation is only performed on the weights of the MINE networks. After training the MINE network for 1000 epochs, the expectations are evaluated to find the estimates of mutual information on the information plane. Therefore, each dot on the information plane of the *i*-th layer represents the estimates of the MINE networks for $I(X;\;h_{i})$ and $I(h_{i};Y)$, for a single epoch of VGG-16, after training the MINE network for 1000 epochs. Each mutual information estimate shown on the information plane is obtained by the same number of training iterations. To visualize the information plane, we consider the first 50 epochs of the VGG-16 training phase. The mutual information values do not provide more insight on the compression phase beyond that point. The above procedure is repeated for all 50 VGG-16 epochs shown in the information plane.

**Figure 3:**
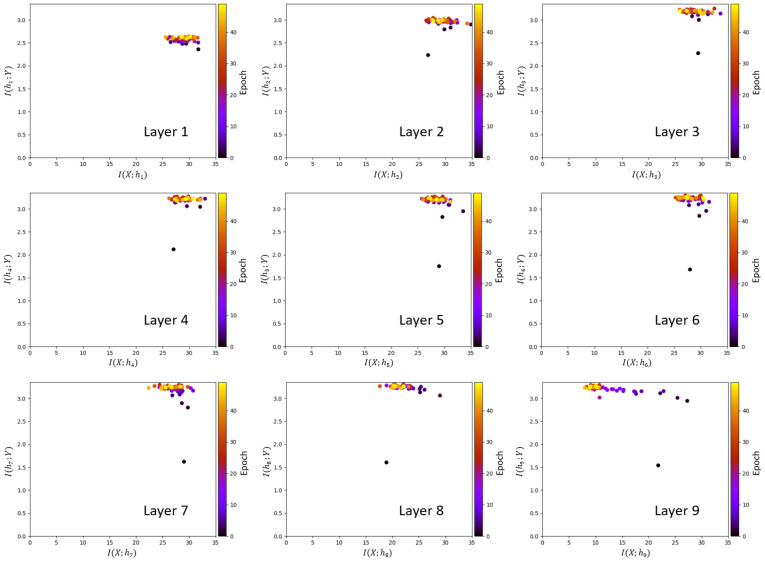

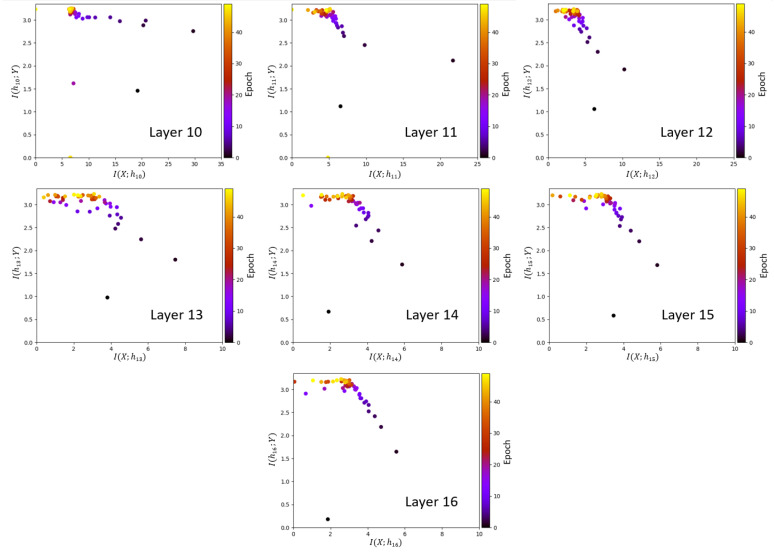
Information planes for the VGG-16 layers trained on Canadian Institute for Advanced Research (CIFAR)-10 image set.

**Figure 4:**
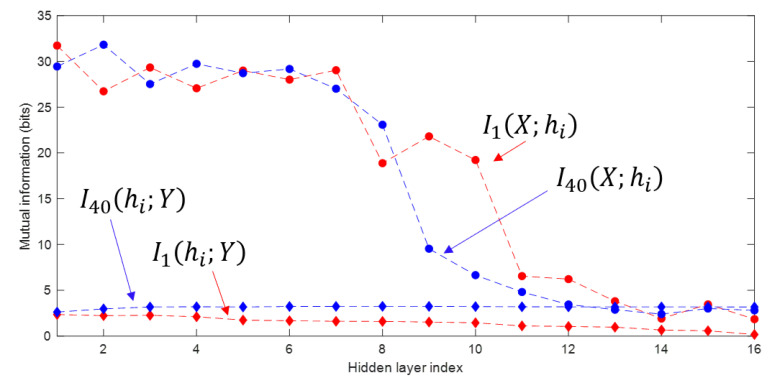
Mutual information estimates as function of the layer index, for the 1st and the 40th epoch. The subscript *n* in $I_{n}\left(h_{i};Y\right)$ indicates the epoch.

The information planes of the VGG-16 layers are shown in Figure 3. The mutual information estimates are expressed in bits. The compression phase is evident especially in the high-order layers, which is consistent with previous work presented in [2], however for the first time shown in a CNN with such a high complexity. One further difference with respect to [2] is that for the VGG-16 network trained on CIFAR-10, the compression phase appears earlier in the training process. We see that $I(X;\;h_{i})$ starts decreasing after the first VGG-16 epoch for the high-order layers and continues to exhibit a decreasing trend until convergence. The estimation of $I(h_{i};Y)$, for all layers i=2,…,16, converges towards the upper bound equal to $\log_{2}10$, which is the desired value of the mutual information in bits as CIFAR-10 contains 10 classes. An exception is constituted by the first layer, which seems to slightly underestimate the mutual information with the output. It can also be seen how the input is compressed successively in each layer. This behavior is more clear from layer 7 onward, as the estimates of $I(X;\;h_{i})$ decrease between subsequent layers, as demanded by the DPI. While training the MINE networks, it was observed that the mutual information estimates converged to zero during training for some VGG-16 epochs. The occurrence of such events was significantly mitigated by lowering the learning rate. The lower learning rate, however, slows down the training process. Therefore, training over 1000 epochs was needed for MINE to allow the mutual information estimates to reliably converge. Figure 4 shows the decrease of the mutual information estimates $I(X;\;h_{i})$ and $I(h_{i};\;Y)$ as a function of the layer index, for the 1st and the 40th epoch, i.e., towards beginning and end of the considered training interval, respectively, thus indicating that the DPI is well approximated by the MINE.

We remark that the results presented in this section are qualitative. Proper quantitative assessment of the variance in the trajectories and a comparative study of the convergence of DNNs having different architectures will be the subject of further investigation.

## 5. Long-Term DNN Regularization

### 5.1. MINE-Based Regularization

Using MINE as a regularizer was proposed in [9] for a small fully connected network trained on MNIST. The authors replaced the variational approximation of the mutual information in [10] with a MINE network for the mutual information estimate. We also consider a MINE network to estimate the mutual information, however with a VGG-16 network of significantly higher complexity. In our experiments, we estimated the mutual information of two layers by applying MINE networks. We trained a VGG-16 network for a total of 10,000 epochs to investigate how the MINE-based regularizer affects the test accuracy over the entire training period. An additional loss term was included in the objective function of the network that represents the estimates of $I(X;\;h_{14})$ and $I(X;\;h_{15})$ by MINE. The network parameters of the MINE networks were the same as described in Section 4.1. We performed gradient descent on the cross-entropy loss with a regularization term that equals the sum of the mutual information estimations of $I(X;\;h_{14})$ and $I(X;\;h_{15})$ multiplied by a regularization coefficient, which was chosen as $\beta =10^{-3}$. The overall loss function is defined as

(12) $$L=L_{CE}+\sum_{l=1}^{L}L_{MINE}\left(l\right)$$

where $L_{CE}$ and $L_{MINE}$ are defined in Equations (6) and (11), respectively, and *L* is the number of layers in the VGG-16 over which the regularization takes place.

The results without and with MINE-based regularizer are shown in Figure 6a,b, respectively. The test accuracy increases and the variance decreases with respect to the experiments without MINE-based regularization. The maximum test accuracy achieved with the MINE-based regularizer is 93.9%, whereas a baseline accuracy for a VGG-16 network is measured as 93.25% in [23], which is similar to our results shown in Figure 6a.

### 5.2. VIB-based Regularization

As an alternative mutual-information-based estimation method between consecutive layers in CNNs, a Variational Information Bottleneck (VIB) method was proposed in [10] for fully connected networks with low complexity. The VIB technique was also used in [24] to reduce network complexity. Here we extend VIB-based regularization to CNNs with substantially higher complexity. We investigate the performance of the regularizer when training a VGG-16 for a large number of training epochs, up to 10,000, in which case overfitting is a common issue.

We adopt the same formulation of the VIB as in [24]. In a feed-forward neural network like the VGG-16 network, each hidden layer, $h_{i}$, takes as input the previous output of the hidden layer, $h_{i-1}$. Therefore, each layer only extracts information from the previous layer. The previous layer typically contains some information that is not relevant to the output. The aim of a VIB-based regularizer is therefore to reduce the amount of redundant information extracted from the previous layer. This is accomplished by minimizing the estimated mutual information between subsequent layers, $I(h_{i};\;h_{i-1})$. The information bottleneck objective then becomes

(13) $$L=\sum_{i=1}^{L}\beta_{i}I\left(h_{i};\;h_{i-1}\right)-I\left(h_{i};\;Y\right)$$

where the coefficient $\beta_{i}\geq 0$ represents the strength of the VIB-based regularization in the *i*-th layer, and *L* is the number of layers in the network over which the regularization takes place. An upper bound on the term given by Equation (13) can be derived as

(14) $$\hat{L}=\sum_{i=1}^{L}\beta_{i}E_{h_{i-1}∼p\left(h_{i-1}\right)}\left[D_{KL}\left[p\left(h_{i}|h_{i-1}\right)∥\;q\left(h_{i}\right)\right]\right]-E_{x,y∼D}\left[\log\;q\left(y|h_{L}\right)\right]$$

where D denotes the input data distribution and $q(h_{i})$ and $q(y|h_{L})$ are variational distributions that approximate $p(h_{i})$ and $p(y|h_{L})$, respectively. To optimize the network, a parametric form of the distributions $p(h_{i}|h_{i-1})$, $q(h_{i})$ and $q(y|h_{L})$ is specified. In [24], it is assumed that each conditional distribution $p(h_{i}|h_{i-1})$ is defined via the relation

(15) $$h_{i}=\left(\mu_{i}+\epsilon_{i}⊙\;\sigma_{i}\right)⊙f_{i}\left(h_{i-1}\right)$$

where the parameters $\mu_{i}$ and $\sigma_{i}$ are learnable for each layer where VIB is applied and $\epsilon_{i}\;∼N\left(0,I\right)$. The function $f_{i}$ represents the network processing that takes place at the *i*-th layer, consisting of a linear transformation or a convolution operation for convolutional layers, plus batch normalization and a non-linear activation function. Furthermore, the distribution $q(h_{i})$ is specified as a Gaussian distribution, such that

(16) $$q\left(h_{i}\right)=N\left(h_{i};\;0,\mathrm{diag}\left[\xi_{i}\right]\right)$$

where $\xi_{i}$ is a vector of variances learned from the data.

**Figure 5:**
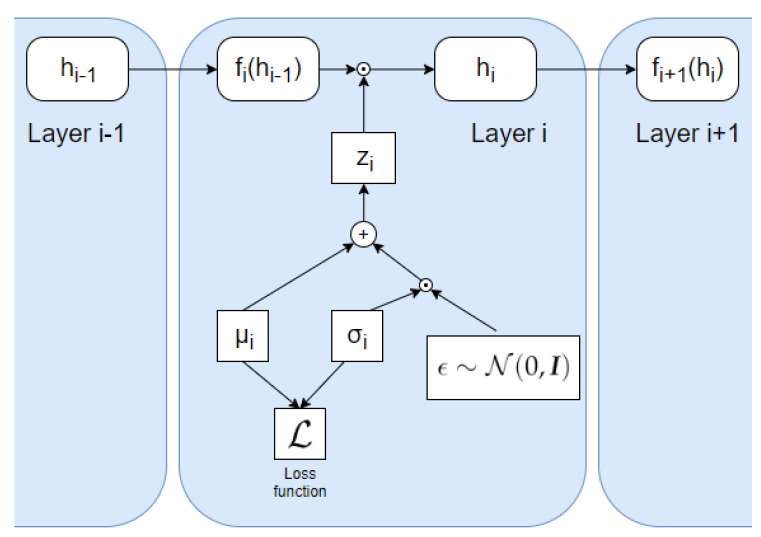
Illustration of how the VIB is incorporated into each layer using the formulation from [24] using Equation (15), where $z_{i}=\mu_{i}+\epsilon_{i}⊙\;\sigma_{i}$. The noise variable, $\epsilon_{i}$, is sampled randomly from a Gaussian distribution with zero mean and unit variance.

The process is illustrated in Figure 5. The element-wise multiplications in Equation (15) are applied differently in fully connected layers and convolutional layers, as the convolutional layers have several channels. For each convolutional layer the learned parameters, $\mu_{i}$ and $\sigma_{i}$, are vectors with dimensionality equal to the number of channels in the layer. Therefore, we obtain a learned Gaussian distribution for each channel. The matrix, which is adopted for the element-wise multiplications, has the same dimensions as the convolutional layer output, and is generated by sampling from the distribution of each channel $n^{2}$ times, where n×n is the feature map size. For the fully connected layers, the vectors of the learned parameters have a dimensionality equal to the number of neurons in the layer. Accordingly, each element is associated with a separate learned Gaussian distribution. Thus, the loss function is expressed as

(17) $$\hat{L}=\gamma \sum_{i=1}^{L}\beta_{i}\sum_{j=1}^{r_{i}}\log\;\left(1+\frac{\mu_{i,j}^{2}}{\sigma_{i,j}^{2}}\right)-E_{x,y∼D}\left[\log\;q\left(y|h_{L}\right)\right]$$

where $r_{i}$ denotes the number of channels for convolutional layers and neurons for fully connected layers. The coefficient γ is used to scale the regularizing term. Scaling is crucial in deep networks as the accumulated loss from every layer may otherwise become too large.

As in the previous sections, the network adopted in our experiments was a VGG-16, trained on the CIFAR-10 dataset with the same data augmentation described in Section 4. The regularization constants were chosen as $\gamma =10^{-5}$, ${\left\{\beta_{i}\right\}}_{i=1,\ldots ,15}=\{2^{-5},2^{-5},2^{-4},2^{-4},2^{-3},2^{-3},2^{-3},2^{-2},2^{-2},2^{-2},$ $2^{-1},2^{-1},2^{-1},1,1\}$.

**Figure 6:**
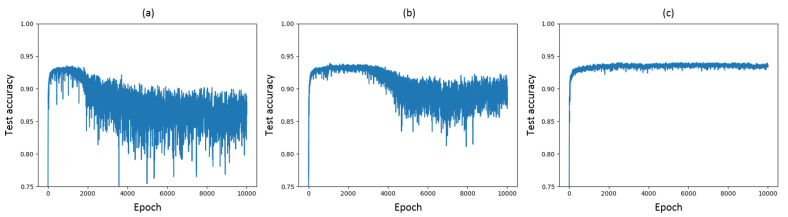
Test accuracies over 10,000 epochs for VGG-16 trained on CIFAR-10 (**a**) without mutual- information-based regularizer, (**b**) with MINE-based regularizer and (**c**) with Variational Information Bottleneck (VIB)-based regularizer.

We trained the VGG-16 network without and with the VIB objective and compared the results. The output of the i-th layer in the experiment with the VIB was calculated as shown in Equation (15). Both models were trained for a total of 10,000 epochs on the CIFAR-10 dataset. To update the weights by back-propagation, we used the Adam [25] optimizer with exponential decay rate parameters $\beta_{A,1}=0.9$, $\beta_{A,2}=0.999$ and $\epsilon_{A}=10^{-8}$. The learning rate was fixed to 0.001. We trained all models using a mini-batch size of 128. The results without and with the VIB objective are illustrated in Figure 6a,c, respectively.

The results in Figure 6c show that the test accuracy of the model increases with the additional VIB-based regularizer, achieving a value of 94.1%. We recall that the baseline accuracy for a VGG-16 network in [23] is 93.25%. Furthermore, the VIB-based regularizer prevents the model from overfitting. When trained for enough epochs, the model without the VIB-based regularizer eventually starts to overfit, even though it applies several regularization methods such as dropout, batch normalization and data augmentation, see Figure 6a. In contrast, the test accuracy exhibits substantially lower variance if the VIB objective is considered. To obtain the best accuracy from the model trained without VIB, early stopping is required. In contrast, the test accuracy of the model with VIB is much more stable and the stability is maintained even after 10,000 epochs of training.

**Figure 7:**
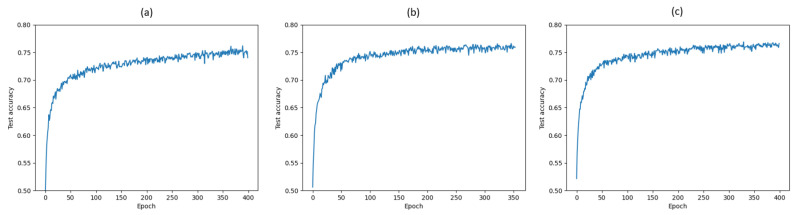
Test accuracies over 400 epochs for LeNet-5 trained on CIFAR-10 (**a**) without mutual-information- based regularizer, (**b**) with MINE-based regularizer and (**c**) with VIB-based regularizer.

We remark that the results relative to Figure 6a,b are obtained by using the exact same VGG-16 network architecture depicted in Figure 1. The VIB block illustrated in Figure 5 is added for regularization into each layer to get the results shown in Figure 6c, resulting in a modification of the loss function as well as of the overall network architecture. An interesting aspect related to the application of VIB for regularization is whether the observed improved performance is due to the modified loss function and architecture, or rather to the injection of noise alone. To investigate this aspect, we resort to a simpler LeNet-5 network on CIFAR-10. First, a comparison of test accuracy over 400 epochs without mutual-information-based regularizer, with MINE-based regularizer including the mutual information estimates of $I(X;\;h_{i})$, i=1,…,4, and with VIB-based regularizer is shown in Figure 7. The regularization coefficients for MINE and VIB are chosen similarly to the case of VGG-16. As observed in the case of VGG-16 on CIFAR-10, a VIB-based regularizer leads to better performance, albeit significantly lower than that achieved by VGG-16, as LeNet-5 is a much simpler network. For the same reason, overfitting is generally not an issue for a LeNet-5 network, and therefore the possible improvements due to regularization are marginal. Second, the performance of a VIB-based regularizer for LeNet-5 is compared with that achieved by a network where a Gaussian noise signal with zero mean and fixed standard deviation is added at each hidden layer. The accuracy obtained after 400 training epochs is reported in Table 1 for various values of the Gaussian noise standard deviation σ, and compared with that achieved by either VIB, MINE, or no regularization. It appears that the addition of noise alone is not adequate to achieve the same performance as the other regularizers.

## 6. Conclusions

Information theoretic concepts were adopted to analyze and improve high-complexity CNNs. We demonstrated the convergence of mutual information on the information plane and the existence of a compression phase for VGG-16, thus extending the results of [1] for fully connected networks with low-complexity. Furthermore, our experiments highlighted the advantages of regularizing DNNs by mutual-information-based additional terms in the network loss function. Specifically, mutual- information-based regularization improves and stabilizes the test accuracy, significantly reduces its variance, and prevents the model from overfitting, especially for a large number of training epochs.

## Figures and Tables

**Table 1:** Test accuracies for LeNet-5 trained on CIFAR-10 with various regularization methods.

Regularization method	VIB	MINE	none	σ = 0.1	σ = 0.2	σ = 0.3	σ = 0.4
Accuracy (%)	76.9	76.7	75.9	73.8	73.9	72.3	72.3

## Abbreviations

The following abbreviations are used in this manuscript:

DNN	Deep Neural Network
CNN	Convolutional Neural Network
MINE	Mutual Information Neural Estimation
ReLU	Rectified Linear Unit
VGG	Visual Geometry Group
IB	Information Bottleneck
KL	Kullback–Leibler
DPI	Data Processing Inequality
CIFAR	Canadian Institute for Advanced Research
CONV	Convolutional
FC	Fully Connected
MaxPool	Maximum Pooling
VIB	Variational Information Bottleneck
